# Comparison of two diagnostic methods through blood and urine sample analyses for the detection of ketosis in cattle

**DOI:** 10.14202/vetworld.2022.737-742

**Published:** 2022-03-26

**Authors:** Karla Verónica Borja, Andrés Miguel Amador, Silvana Hipatia Santander Parra, Cristian Fernando Cárdenas, Luis Fabian Núñez

**Affiliations:** 1 Facultad de Ciencias de la Salud, Carrera de Medicina Veterinaria y Zootecnia; Universidad de Las Américas, Quito, Ecuador, Antigua Vía a Nayón S/N, Quito EC 170124; 2 Facultad de Ciencias de la Salud, Carrera de Medicina Veterinaria y Zootecnia, One Health Research Group; Universidad de Las Américas, Quito, Ecuador, Antigua Vía a Nayón S/N, Quito EC 170124

**Keywords:** acetoacetates, cattle, hydroxybutyrates, ketosis, postpartum period

## Abstract

**Background and Aim:**

Several Ecuadorian farms use human test strips (cheaper than veterinary strips) to diagnose bovine ketosis; however, their reliability is unknown. This study aimed to determine the confidence level of human strips for the detection of ketosis in bovines by comparing two diagnostic methods for ketosis: one used in bovines (gold standard) to analyze blood samples and the other used in humans to analyze urine samples.

**Materials and Methods:**

The study was conducted on an Ecuadorian farm using 50 animals, ten from each of five categories: heifers, 4 months pregnant (4MP), 15 days prepartum (15DPRE), 15 days postpartum (15DPOST), and 42 days postpartum (42DPOST). Blood samples were collected through coccygeal venipuncture and urine samples were collected during spontaneous urination. BHBCheck™ assay was used to measure b-hydroxybutyrate (BHB) in the blood, whereas Combur10Test^®^ was used to measure acetoacetate (AcAc) in urine for the determination of ketosis.

**Results:**

BHB was detected in all animals. Based on a ketosis cutoff point of 0.8-1.2 mmol/L, 13 animals from the 15DPOST and 42DPOST categories had ketosis; AcAc was detected in the urine from nine animals originated from the two same categories. Metabolites, either BHB or AcAc, were not detected in heifers, 4MP, or 15DPRE individuals. Finally, the BHBCheck™ assay had better efficiency in detecting ketosis in animals (p<0.05) than the Combur10Test^®^.

**Conclusion:**

Combur10Test^®^ urine strips reached 92% reliability for the detection of ketosis in dairy cattle, compared to BHBCheck™ assays.

## Introduction

In dairy cattle, ketosis is a common metabolic disease that typically occurs during early lactation; it is characterized by a state of inappetence, and occasionally, signs of nerve dysfunction such as pica disorder, incoordination, and abnormal gait [1-4]. Ketosis is more common during the management of high-yielding dairy cows. Clinical and subclinical ketosis induces a drop in milk production and reproductive capacity. Furthermore, the risk of abomasum displacement is increased due to ketosis-related pathologies and the decline in food intake during ketosis symptoms [5-9].

Genetic evolution has permitted the development of dairy breeds that produce high daily quantities of milk, with Holstein Friesians being the highest milk-producing breed (by volume). Nonetheless, high milk production levels can lead to several functional, reproductive, and metabolic problems, such as ketosis, in cattle [10,11]. Ketosis is diagnosed by assessing the levels of acetoacetate (AcAc) or b-hydroxybutyrate (BHB) in the blood, urine, or milk, respectively. In addition, it can also be diagnosed through the appearance of clinical signs and risk factors [12-14].

In areas with limited access to ketosis diagnostic methods, the measurement and control of urinary ketones have been used as a diagnostic tool. Test strips developed for humans are commonly used because they are cheaper than those intended for animals; however, whether they truly work in animals in addition to their sensitivity and specificity remain unknown.

This study aimed to conduct a comparative study evaluating a specific diagnostic method for ketosis in cattle BHBCheck™ PortaCheck, Inc., Nueva Jersey, USA]), compared to the measurement of ketones in urine as assessed through test strips intended for humans (Combur10 Test^®^), to determine the reliability of both diagnostic methods in dairy cattle, such that veterinarians can obtain accurate results for the detection of ketosis.

## Materials and Methods

### Ethical approval

All procedures conducted in the present investigation were in accordance with the guidelines and the Government Manual for Taking and Sending Samples from Domestic Animals by the Laboratories of the Animal Health Directorate, Animal Resources of the Agency of Regulation and Control of Phytosanitary and Animal Health of Ecuador, under the identification: Instructive INT/DA/019.

### Study period and location

The study was conducted from December 2018 to February 2019. The present study was conducted on an Ecuadorian farm (Hacienda Miraflores Bajo #4) located in the province of Pichincha, Ecuador.

### Animals

The present study was conducted on an Ecuadorian farm (Hacienda Miraflores Bajo #4) located in the province of Pichincha, in Ecuador. The farm has 281 dairy cows from different breeds, with the main breeds being Holstein, Jersey, and Brown Swiss. Animals are managed using a grazing system based on *Lolium perenne* (30%), *Lolium multiflorum* (20%), *Pennisetum clandestinum* (30%), *Dactylis glomerate* (10%), and *Trifolium repens* (10%), whereas highly productive animals (average milk production of approximately 28 L/day) also receive 4 kg of Winavacas (Winavena, Pintag, and Ecuador) food supplement. The cows are protected from diseases by vaccination and regularly inspected by veterinarians.

In the present investigation, cows were classified into six groups according to their physiological status: veal (n=47), heifer (n=73), 4 months pregnant (4MP; n=39), 15 days prepartum (15DPRE; n=38), 15 days postpartum (15DPOST; n=44), and 42 days postpartum (42DPOST; n=40). From these six groups, ten animals were randomly selected from each physiological group based on inclusion and exclusion criteria (Table-1), resulting in the selection of a total of 50 cows for inclusion in this study (excluded veal).

**Table-1:** Inclusion and exclusion criteria.

Inclusion	Exclusion
Heifers 4 months pregnant 15 days prepartum 15 days postpartum 42 days postpartum	Calves Distorted delivery Prepartum and postpartum pathologies

### Sampling and sample processing

For each sample, 1 mL of fresh blood was drawn through venipuncture of the coccygeal vein by lifting the cow’s tail and using non-sterile examination gloves. From the collected sample, 20 mL of blood was placed in the BHBCheck™ device (Portacheck - USA) to determine the ketosis result (either positive or negative), whereby the range of 0.35-0.8 mmol/L was considered a negative result, and values above 0.8 mmol/L were considered a positive result.

A urine sample was collected from the same animal by spontaneous urination after cleaning the vulva area to prevent feces contamination of the sample. Once the urine sample was obtained, 1 mL of urine from the respective sterile container was placed drop-by-drop on a Combur10 Test^®^ strip (F. Hoffmann-La Roche AG, Basel, Switzerland) to evaluate the ketosis status, wherein ≤0.5 mmol/L was considered a negative result and any value greater than this cutoff was considered a positive result (e.g., 1±1 mmol/L; 2±5 mmol/L; and 3±mmol/L). Both procedures were repeated for each of the 50 cows belonging to the five different categories.

The ketone body results were interpreted according to the protocol guidelines of the diagnostic tests (BHBCheck™ for blood and Combur10 Test^®^ for urine). The results of the BHBCheck™ test were produced immediately after placing the blood sample, whereas the results of the Combur10 Test^®^ strips were verified against the corresponding colorimetric scale.

All procedures conducted in the present investigation were in accordance with the guidelines and the Government Manual for Taking and Sending Samples from Domestic Animals by the Laboratories of the Animal Health Directorate, Animal Resources of the Agency of Regulation and Control of Phytosanitary and Animal Health of Ecuador, under the identification: Instructive INT/DA/019.

### Statistical analysis

The obtained results were statistically analyzed using the IBM statistical package for the social sciences^®^ Statistics 25.0 (IBM Corp., NY: IBM Corp). Descriptive statistics were compiled, wherein measures of central tendency were determined for the values obtained from the blood samples, and a Shapiro–Wilk test was applied to verify a normal distribution for each category. Subsequently, the BHB results from the blood of all study groups were compared using an analysis of variance at a 95% confidence threshold. Afterward, Duncan’s test was applied, which works with a changing mean threshold. Due to the mostly non-normally distributed data, a Kruskal–Wallis test was applied to compare the AcAc levels across the five categories of sampled animals, to verify whether there was a significant difference in the distribution of the values in urine; statistical significance was set at p<0.05. With respect to the entire population comparison of BHB in blood and AcAc in urine, a Wilcoxon signed-rank test with a 95% confidence level was performed to determine whether the two tests were equally effective in detecting ketosis. The specificity and sensitivity of the Combur10 Test^®^ were also calculated [15].

## Results

**Figure-1:**
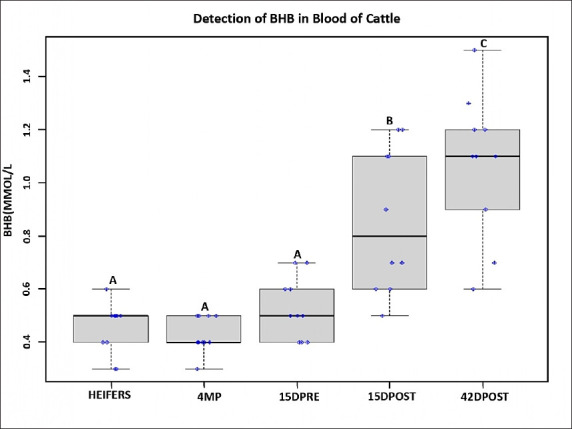
Detection of β-hydroxybutyrate in blood of cattle.

With respect to the blood sample results, the BHB levels of heifers, 4MP, and 15 DPRE cows followed a normal distribution forming a suitable Gaussian bell, illustrating a negative diagnosis for ketosis. Conversely, in the 15DPOST group, five cows were positive for ketosis (5/10), whereas in the 42DPOST group, eight cows were positive for ketosis (8/10; Figure-1). There was a statistically significant difference in the BHB values obtained in the blood samples of all categories (p<0.05 [0.000]). The BHB levels of heifers, 4MP, and 15DPRE were similar (p>0.05), with no animal being positive for ketosis. For the other categories, the Duncan test showed that the mean BHB level of 42DPOST individuals was higher (p<0.05) than that of 15DPOST individuals, which was, in turn, higher (p<0.05) than those of the three other categories; animals in both the 42DPOST and 15DPOST categories were positive for ketosis (Table-2 and Figure-1). This revealed that ketosis was present solely in cows from the 15DPOST and 42DPOST groups.

**Table-2:** Duncan’s test and means subsets formation between the means of blood values of BHB.

	Category	N	A	B	C
Duncan^a^	4MP	10	0.430		
	Heifer	10	0.450		
	15DPRE	10	0.530		
	15DPOST	10		0.860	
	42DPOST	10			1,070
	Sig.		0.266	1,000	1,000

a Uses the sample size of the harmonic mean = 10,000; 4MP = 4 months pregnant; 15DPRE = 15 days prepartum; 15DPOST = 15 days postpartum; 42DPOST = 42 days postpartum; Sig=Significance.

**Figure-2:**
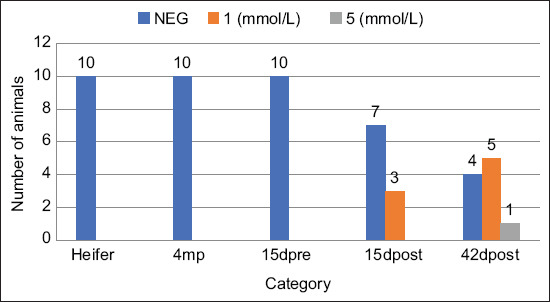
Detection of acetoacetate in urine of cattle.

For the urine samples, 41 (82%) of all animals were negative for ketosis, with the remaining 9 (18%) being positive for the disease. Among the animals with positive results, 8 (16%) produced urine with 1 mmol/L of AcAc and 1 (2%) produced urine with 5 mmol/L of AcAc. In terms of their AcAc values, all heifers, 4MP, and 15DPRE animals (100%) were negative for ketosis. Conversely, in the 15DPOST category, 70% (7/10) of the animals were negative for ketosis, whereas 30% (3/10) were positive, with a value of 1 mmol/L of AcAc in their urine. In the 42DPOST category, 40% (4/10) of the animals were negative for ketosis, whereas 50% (5/10) were positive with 1 mmol/L of AcAc in their urine and 10% (1/10) were positive with 5 mmol/L of AcAc in their urine (Figure-2).

The Kruskal–Wallis test revealed that the urinary AcAc values differed across categories (p<0.01). Moreover, the blood BHB and urinary AcAc levels differed between the two diagnostic methods when applied to the 50 bovines in this study (p<0.05). The results for the BHB (mmol/L) levels in blood were obtained using the BHBCheck™ gold standard test, intended for animal use, whereas the results of the AcAc (mmol/L) levels in urine were obtained using the Combur10Test^®^ intended for humans. To obtain the true positive and true negative results between the two diagnostic methods for ketosis, the values in urine that did not concur with the values in blood were counted to provide a level of confidence for the use of test strips intended for humans for the diagnosis of ketosis in dairy cattle (Table-3).

**Table-3:** Results for ketosis in blood and urine samples from the 50 animals sampled.

Results of blood test		Results of urine test	
	
Positives	Negatives	Positives	Negatives
13	37	9	41

Of the 50 animals sampled, the results of four animals were false negatives according to the urine test. Therefore, of the animals that were subjected to ketosis diagnosis using the Combur10 Test^®^, 8% produced different results from that achieved based on the measurement of BHB, whereas the results of the remaining 92% were reliable. The Combur10 Test^®^ showed a test specificity of 100% and sensitivity of 69.23%.

## Discussion

In this study, two diagnostic methods were evaluated to detect ketosis based on blood and urine samples. Consequently, the results depict that, across categories, the BHB values in blood (mmol/L) differed across groups, producing three mean subsets: Group A encompassed averages of 0.430 mmol/L (4MP), 0.450 mmol/L (heifers), and 0.530 mmol/L (15DPRE); Group B had an average of 0.860 mmol/L (15DPOST); and Group C had an average of 1.070 mmol/L (42DPOST). These results agree with those of a study published by Oetzel [1], in which the cutoff point for subclinical ketosis in a bovine was 0.8 mmol/L. The average of subset B is consistent with this cutoff (corresponding to the 15DPOST category) as is the average of subset C (42DPOST), presenting a higher degree of ketosis without becoming clinical. Oetzel [1] indicates that the cutoff point for clinical ketosis is 1.6 mmol/L. On the other hand, Sailer *et al*. [16] suggested that 1.2 mmol/L should be used as the cutoff for the diagnosis of hyperketonemia without discriminating subclinical from clinical ketosis.

In the present investigation, there were differences in the measurements of ketone bodies in the samples analyzed using the two diagnostic methods. BHB was evaluated in blood and AcAc was evaluated in urine. This difference may be explained by the concentration and circulation of this metabolite in the corporal fluids of cows [17,18].

Horber *et al*. [19] conducted a study in which the levels of BHB and AcAc in both blood and urine from healthy and ketotic cows were compared using a laboratory diagnostic method. In healthy cows, the blood values of AcAc and BHB were 0.06 and 0.3 mmol/L, respectively, whereas the urine values of AcAc and BHB were 0.8 and 0.2 mmol/L, respectively. In ketotic cows, the blood values of AcAc and BHB were 0.4 and 1.2 mmol/L, respectively, whereas the urine values were 9.4 and 7 mmol/L, respectively [19]. According to laboratory tests, these results complemented those of Schultz [20], which indicated that, in a clinical state of ketosis, the AcAc level in urine is 4 times greater than the BHB level in blood. These proportions were not obtained in the present study since the BHB and AcAc levels were compared using different samples and the results were interpreted in the field, in contrast to the aforementioned investigations in which the same ketone body was compared in different samples and results were analyzed in the laboratory using different techniques.

Työppönen and Kauppinen [21] stated that the most stable ketone body for the diagnosis of ketosis in bovines is BHB. Keltanen [22] affirmed that AcAc is the first ketone body to be formed and is subsequently reduced into BHB by the enzyme BHB dehydrogenase; this repeatedly occurs as the formation of ketone bodies is part of the tricarboxylic acid cycle. The current investigation results agree with this process: in 4 out of 50 cows, the ketone body AcAc measured in urine was different from the blood measurement of the BHB ketone body, confirming the stability of BHB in blood and the volatility of AcAc in urine.

Sailer *et al*. [16] compared the sensitivity and specificity of two ketosis diagnostic methods (Precision Xtra™ and BHBCheck™) through the detection of BHB in blood; and the Precision Xtra™ meter had a sensitivity of 98% and a specificity of 92%, whereas the BHBCheck™ meter had a sensitivity of 91% and a specificity of 93%. In the present study, BHBCheck™ was used, which has a high specificity, allowing for the detection of true positive results; it is considered a gold standard test. In addition, Oetzel [1] investigated the sensitivity and specificity of diagnostic tests for ketosis using blood and urine samples from bovines: in blood, the sensitivity was 98% and specificity was 92%, whereas, in urine, sensitivity was 70% and specificity was 97%. In addition, Oetzel [1] noted that collecting a urine sample is more complicated than collecting a blood sample, making it more challenging to apply the diagnostic tool. In the current investigation, 92% of the results of urine samples were similar to those obtained from blood, with the remaining 8% (4 of 50) producing urine levels that did not match those from blood samples; this may be due to the low sensitivity of the colorimetric test strips in diagnosing urine ketosis.

Herein, the Combur10 Test^®^ kit for humans was used, which had reliability of 92%, with the remaining 8% of the results not coinciding with those of the gold standard test; this may have occurred because the kit is intended for human use. Vuljanic *et al*. [23] noted that Combur10 Test^®^ strips had a sensitivity of 97% and a specificity of 81% for glucose; these values may be similar for ketone bodies, since the test’s sensitivity and specificity have not been studied. However, in relation to ketone bodies, Stodulska [24] indicated that, when using the Combina Urine Test Strips to diagnose ketosis in humans, the analytical sensitivity was 98% and the analytical specificity was 97% measured with the minimum amount of 0.15 mmol/L of AcAc. Similarly, a study by Passato [25] affirmed that the Combina 10 HUMAN test strips are a useful tool for diagnosing ketosis in cattle when measuring urinary AcAc levels. The Combur10 Test^®^ tool for humans can be extrapolated to veterinary medicine for the diagnosis of ketosis in bovines, given that its reliability level was 92%, with an 8% error range (false negatives, 4/50), compared with the BHBCheck™ gold standard test.

In this study, the circulation and volatility of ketone bodies were different for BHB in blood and AcAc in urine; this is a consequence of the stability of each ketone body, with BHB being more stable in blood than AcAc and AcAc being more stable in urine than BHB [26,27]. We used a cutoff point of 0.8-1.2 mmol/L to diagnose ketosis in bovines, considering that the cutoff points depend on equipment calibration and the commercial brand used. In addition, we set these cutoffs due to the sensitivity and specificity of each BHB blood meter, being 91% and 93%, respectively, for BHBCheck™, which does not interfere with its gold standard test characteristic [16].

One of the main limitations of this study was the population distribution across the different categories according to their physiological state; this made it impossible to have animals available for sampling at the same time and place, particularly given the farm’s low pregnancy rate and the lack of farm workers. Moreover, with respect to urine sample collection, certain animals showed discomfort at the time of stimulation to induce spontaneous urination and were reluctant to urinate, making harvesting their urine samples complicated.

## Conclusion

This study compared the BHBCheck™ and Combur 10 Test^®^, finding that both were suitable for ketosis detection in 15DPOST and 42DPOST cows, illustrating that their physiological statuses are in a good period for ketosis diagnosis. Nonetheless, the Combur 10 Test^®^ had a 92% reliability for detecting ketonic bodies through the AcAc detection method, wherein it indicated false negatives. Additional studies that aim to detect ketonic bodies in a herd suspected of ketosis before and after applying treatment are needed to evaluate trends in the formation and elimination of these metabolites using different diagnostic methods [28-31]; this would permit a better analysis of their circulation and levels, which could be used together with animal behavior and clinical signs [4] and allow for the use of a cheaper, more field-appropriate detection test for ketosis in dairy cattle.

## Authors’ Contributions

KVB and AMA: Participated in the experimental work. KVB, LFN, SHSP, and CFC: Analyzed data and results. KVB: Wrote the manuscript. SHSP: Revised manuscript. CFC: Conceptualized and designed the study. LFN: Edited and finalized the manuscript. All authors read and approved the final manuscript.
